# Supplementary figures and images for: Indigo alleviates psoriasis through the AhR/NF-κB signaling pathway: an in vitro and in vivo study
Source: PeerJ. 2024 Oct 21;12:e18326. doi: 10.7717/peerj.18326 (PMC11505883; doi:10.7717/peerj.18326)

The marker we used is Servicebio® Western Protein Marker I, catalog number G2086.

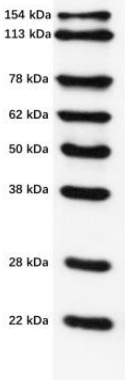

Fig 4C

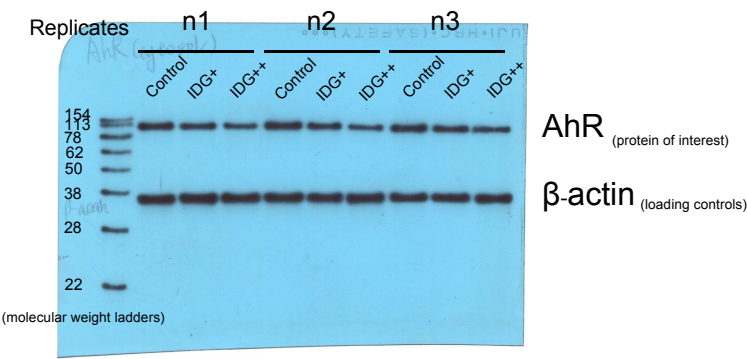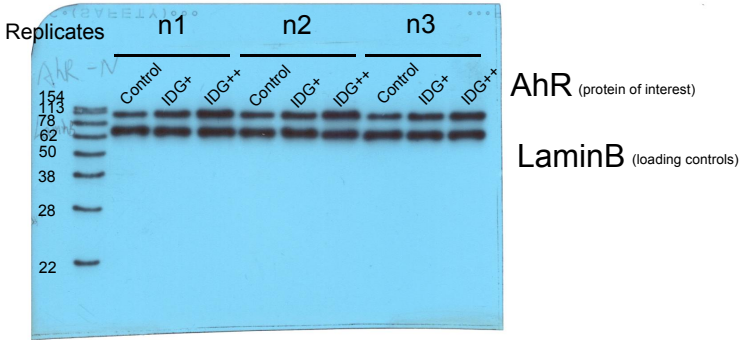

Fig 5

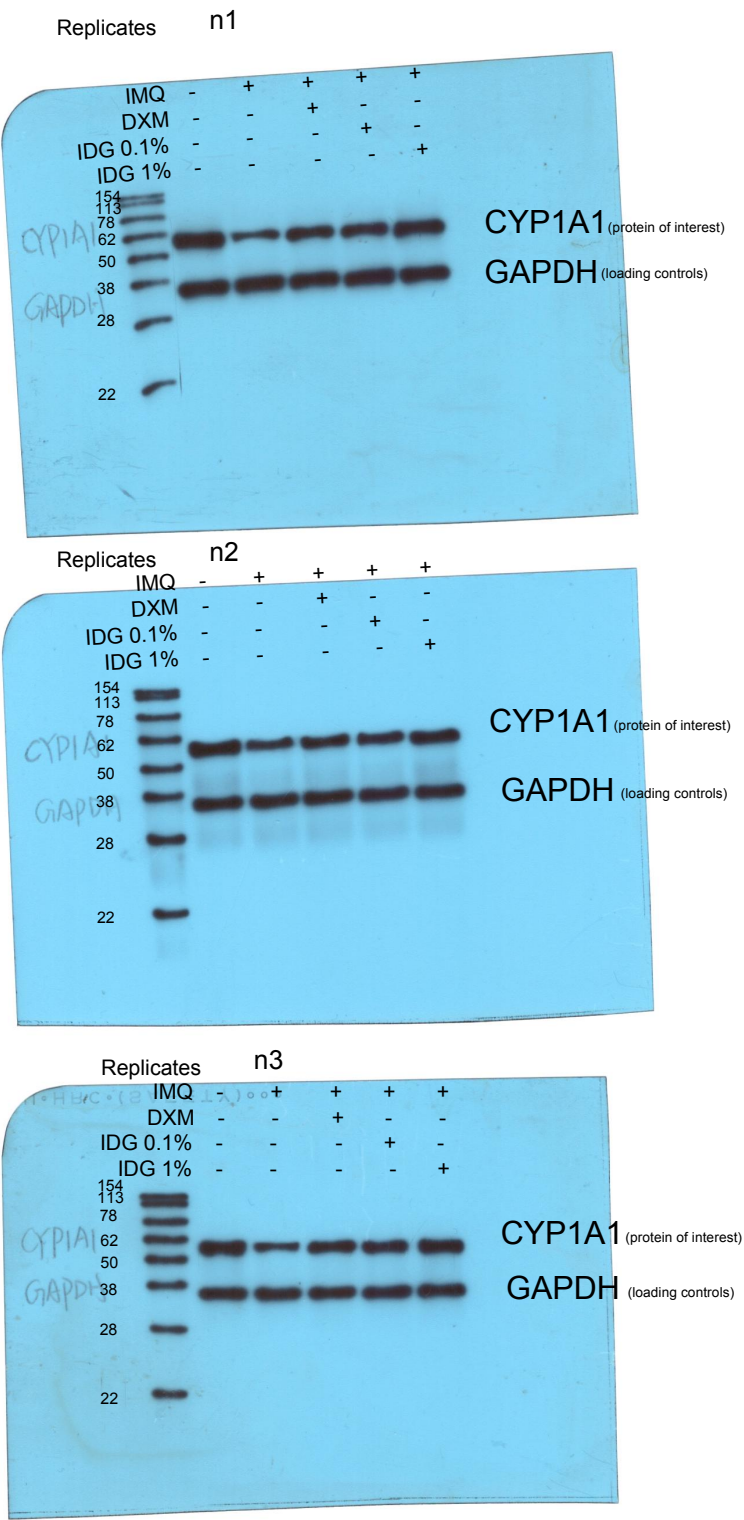

Fig 6

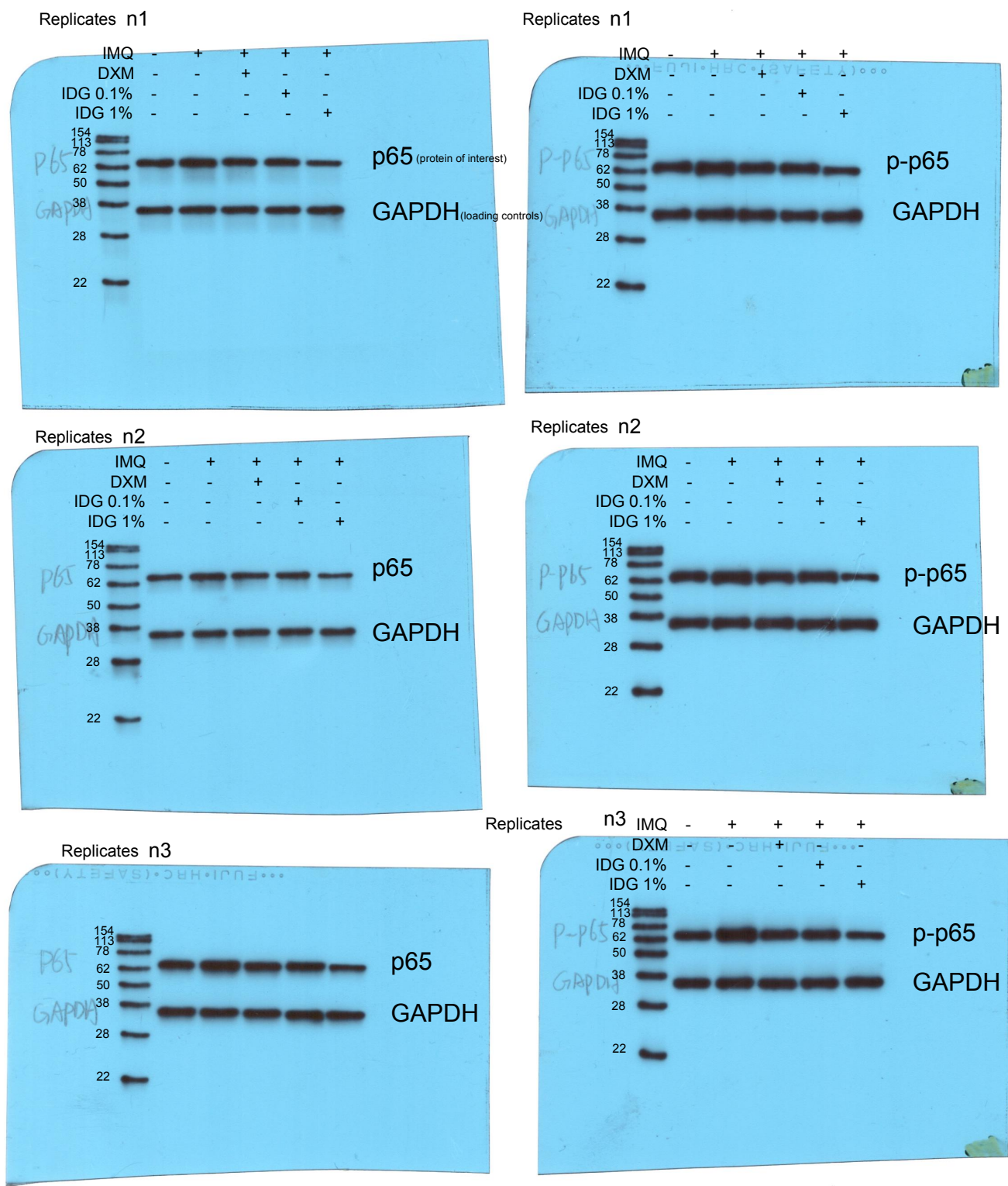

Supplement: Supplemental Information 3 [file peerj-12-18326-s003.pdf]
